# Antibacterial Activity of Leaves of *Cadaba trifoliata* (Roxb.) Wt. & Arn

**DOI:** 10.4103/0250-474X.54271

**Published:** 2009

**Authors:** R. Mythreyi, E. Sasikala, A. Geetha, V. Madhavan

**Affiliations:** Department of Pharmacognosy, M. S. Ramaiah College of Pharmacy, Bangalore-560 054, India; 1 Department of Central Research Institute for Siddha, Chennai-600 106, India; 2 Department of Bharathi Women's College (Autonomous), Chennai-600 108, India

**Keywords:** Antimicrobial activity, *C. trifoliata*, disc diffusion method, pathogenic organisms

## Abstract

Antibacterial activity of aqueous and ethanol leaf extracts of *Cadaba trifoliata* was evaluated by cup plate method against bacterial strains such as *Staphylococcus aureus, Bacillus subtilis, Acinetobacter, Enterobacter aerogenes, Erwinia* and *Escherichia coli*. The ethanol extract of the leaves demonstrated a high degree of activity against all the tested bacterial strains except *Erwinia* and *Acinetobacter,* whereas the aqueous extract of the leaves showed moderate activity against *E. coli, B. subtilis* and *Staph. aureus* and *Enterobacter aerogenes.*

*Cadaba trifoliata* (Capparaceae) is an unarmed branched shrub up to 3 m height. Leaves are palmately trifoliate and the leaflets are oblong or lanceolate[1]. It is locally called as *Manudukurundu* or *Mara viluthi* (Tamil) and possesses antirheumatic, emmenagogue, anthelmintic, antiphlogistic, antisyphilic and antibacterial properties[2,3]. This study was aimed to evaluate the inhibitory effect of aqueous and alcoholic extracts of leaves of *C. trifoliata* against certain bacterial strains.

Leaves of *C. trifoliata* were collected and identified in the Department of Pharmacognosy, Central Research Institute (Siddha) Chennai and a voucher specimen was deposited in the Department of Pharmacognosy, M. S. Ramaiah College of Pharmacy, Bangalore-54. Leaves were shade dried, pulverized into 40 # mesh size. Powdered leaves (50 g) were subjected to exhaustive extraction with 200 ml of alcohol in a soxhlet apparatus. Solvent was removed under vacuum and concentrated to a semisolid residue (yield 7.04% w/w). The marc obtained from the above extraction was subjected to maceration with distilled water for 24 h. It was then evaporated to dryness to get a semisolid residue (yield 8% w/w). Phytochemical screenings of aqueous and alcoholic extracts were carried out[4].

*Staphylococcus aureus, Bacillus subtilis, Acinetobacter, Enterobacter aerogenes, Erwinia* and *Escherichia coli* were subcultured from the stock culture 24 h prior to the experiment in nutrient agar media and used for the study. Stock cultures of all these organisms were obtained from Central Drug Research Institute, Lucknow. Ciprofloxacin was collected as gift sample from Hindustan antibiotics, Pune.

Aqueous and alcoholic extracts of the leaves of *C. trifoliata* were tested for antibacterial activity by cup plate method[5–7]. Nutrient agar media are prepared and sterilized in an autoclave and 10 ml transferred to previously sterilized petriplates. After solidification petriplates were inoculated with *Staphylococcus aureus, Bacillus subtilis, Acinetobacter, Enterobacter aerogenes, Erwinia* and *Escherichia coli* under aseptic conditions. Ciprofloxacin was used as standard drug at a concentration of 100 µg/well. Using a sterile borer, four wells were made and 0.1ml of the control vehicle (sterile distilled water), test drug (200 µg and 800 µg) and standard compound (100 µg) were poured aseptically into the wells. They were incubated at 37° for 24 h. The zone of inhibition was measured using a metric ruler.

Phytochemical screening of the alcoholic extract indicated the presence of alkaloids, tannins, glycosides, steroids and flavonoids. Phytochemical screening of the aqueous extract revealed the presence of glycosides, phenolic compounds, tannins and steroids.

Antimicrobial activity of two different concentrations of extracts of *C. trifoliata* has been evaluated *in vitro* against gram positive and negative bacteria (Table 1) that are known to cause infections in humans and plants. As presented in Table 1 the inhibitory effect of the extracts of *C. trifoliata* was increased by the concentration of extracts. Although both the extracts showed significant inhibitory effect against *Staphylococcus aureus, Bacillus subtilis, Enterobacter aerogenes* and *Escherichia coli* at both concentrations (200 µg/ml and 800 µg/ml) they did not show any activity against *Erwinia* and *Acinetobacter.*

**TABLE 1:** ANTIBACTERIAL ACTIVITY OF LEAVES OF *C. TRIFLOLIATA*

Extracts	Diameter of the inhibitory zone (mm)						
	
	Conc (μg/ml)	*Acinetobacter*	*E. coli*	*B. subtilis*	*Staph. aureus*	*Enterobacter*	*Erwinia*
Aqueous	200	-	14.5	11.6	13.4	14.5	-
Aqueous	800	-	20.2	20.2	17.3	19.8	-
Alcoholic	200	-	16.3	19.5	11	15.2	-
Alcoholic	800	-	21	20.2	20	17	-
Cipro	100	15	27	23	23.5	24	18

* All the values are mean of triplicates. No inhibition zone is denoted by ‘-’. Cipro stands for ciprofloxacin

In conclusion, the aqueous and alcoholic extracts of *C. trifoliata* leaf possess significant inhibitory effect against the tested pathogens. The results obtained were comparable with those of standard drug ciprofloxacin. However both the extracts did not show any inhibitory effect on plant pathogens. The results of the study support the folklore claim of this plant.
